# Efficient Decellularization by Application of Moderate High Hydrostatic Pressure with Supercooling Pretreatment

**DOI:** 10.3390/mi12121486

**Published:** 2021-11-30

**Authors:** Daiki Zemmyo, Masashi Yamamoto, Shogo Miyata

**Affiliations:** 1 Graduate School of Science and Technology, Keio University, 3-14-1 Hiyoshi, Yokohama 223-8522, Japan; nekonigohan@a6.keio.jp (D.Z.); ironmasashi3434@a3.keio.jp (M.Y.); 2 Department of Mechanical Engineering, Faculty of Science and Technology, Keio University, 3-14-1 Hiyoshi, Yokohama 223-8522, Japan

**Keywords:** decellularization, high hydrostatic pressure, cyclic hydrostatic pressure, supercooling, collagen structure, preservation of structure

## Abstract

Decellularized tissues are considered superior scaffolds for cell cultures, preserving the microstructure of native tissues and delivering many kinds of cytokines. High hydrostatic pressure (HHP) treatment could remove cells physically from biological tissues rather than chemical methods. However, there are some risks of inducing destruction or denaturation of extracellular matrices (ECMs) at an ultrahigh level of HHP. Therefore, efficient decellularization using moderate HHP is required to remove almost all cells simultaneously to suppress tissue damage. In this study, we proposed a novel decellularization method using a moderate HHP with supercooling pretreatment. To validate the decellularization method, a supercooling device was developed to incubate human dermal fibroblasts or collagen gels in a supercooled state. The cell suspension and collagen gels were subjected to 100, 150, and 200 MPa of HHP after supercooling pretreatment, respectively. After applying HHP, the viability and morphology of the cells and the collagen network structure of the gels were evaluated. The viability of cells decreased dramatically after HHP application with supercooling pretreatment, whereas the microstructures of collagen gels were preserved and cell adhesivity was retained after HHP application. In conclusion, it was revealed that supercooling pretreatment promoted the denaturation of the cell membrane to improve the efficacy of decellularization using static application of moderate HHP. Furthermore, it was demonstrated that the HHP with supercooling pretreatment did not degenerate and damage the microstructure in collagen gels.

## 1. Introduction

Tissue engineering is a therapeutic approach that aims to regenerate tissues and organs using living cells and three-dimensional scaffolds. Tissue engineering methods reconstruct organs and biological tissues from cells, scaffold materials, and cytokines [1]. Cells adhere to scaffold materials to promote matrix synthesis and tissue reconstruction. Bioabsorbable polymers are usually used as scaffold materials, and they are classified into natural polymers and bioabsorbable synthetic polymers. For natural polymers, collagen, gelatin, and polypeptides are often used in hydrogel or porous materials [2,3,4]. Synthetic polymers include poly L-lactic acid (PLLA), polycaprolactone (PCL), polyglycolic acid (PGA), and poly-lactic-glycolic acid (PLGA) [5,6,7,8]. Natural polymers are superior in cell proliferation and extracellular matrix (ECM) production because they contain cell adhesion molecules, whereas their mechanical strength is not sufficient as a scaffold material for three-dimensional cell cultures. In contrast, synthetic polymers have superior mechanical strength and are useful for controlling their geometric shape, whereas these kinds of polymers do not activate cells as biologically derived scaffolds [9,10]. In addition, neither of these polymers can replicate the complex structures of organs and living tissues.

In recent years, decellularized tissues have been generated by removing cells from biological tissues and organs of humans or other animals [11,12]. Compared to other scaffold materials, decellularized tissues and organs have advantages in utilizing the structure and composition of “natural” three-dimensional ECMs of tissues containing many cytokines [13,14]. Recently, decellularized amniotic membranes have been clinically applied as wound dressings [15]. To manufacture decellularized tissues, cells in living tissues are removed by suppressing the destruction and denaturation of tissues [13]. Decellularization is performed using chemical or physical methods [16,17,18]. In chemical methods, a surfactant is usually used to solubilize the membrane of cells and nuclei [19]. However, the chemical decellularization methods have risks to destroy or denature the structures of ECMs in tissue, and there is also concern about the toxicity of surfactant remaining in the decellularized tissue [20].

Instead of chemical methods, physical decellularization methods have been established; freeze–thawing [21,22,23], electroporation [24,25,26], and application of high hydrostatic pressure (HHP). In this study, we focused on HHP applications for the physical decellularization method. An HHP application method to denature and destroy the cell membrane has been studied to reduce the usage amounts of surfactants and to generate large-sized decellularized tissues. This method eliminates the risk of surfactant remaining caused by chemical decellularization method and has the possibility to retain the structures of ECMs. Several reports have described decellularization of biological tissues by applying HHP. Ultra-HHP application at 980 MPa for 10 min was imposed to decellularize the porcine carotid artery and rat uterine tissue. The decellularized tissues could be used for functional scaffold materials after transplantation in vivo [27,28]. However, ultra-HHP application has a risk of denaturing and damaging native ECM structures [29]. It has also been reported that there was a relationship between the magnitude of HHP and denaturation of collagen. It has been demonstrated that pressures above 320 MPa increase the enthalpy and destabilize the collagen [30]. In our previous study, we reported that the decellularization effect was realized by cyclic application of moderate HHP [31]. However, in our previous study, the cell removal efficiency of our cyclic HHP application method was insufficient for clinical applications. Moreover, there was a risk that the cyclic application of HHP would reduce the lifetime of the pressure vessel for HHP-based decellularization.

In this study, we aimed to preserve the microstructure of the ECM, while maintaining a better decellularization effect as compared to previous studies by moderate HHP static application with pretreatment, so as to denature and weaken the cell membrane. In this study, we focused on supercooling treatment for the denaturation of cell membranes. Supercooling is usually used for cell and tissue preservation to avoid destruction and denaturation of tissue structures. For cell preservation, cryoprotectants are usually added to prevent denaturation of cells and tissues. In contrast, our previous study reported that the cell membrane was denatured with a decrease in cell viability using the supercooling treatment without cryoprotectant. The purpose of this study is to establish a novel decellularization methodology using moderate and static HHP application combined with the supercooling pretreatment. The physical decellularization consists of several processes: denaturation of cell membrane to cause cell death and destruction, preservation of microstructure of biological tissue, and washing process by perfusion or agitation to exclude cell debris from tissues [21,23]. Therefore, we focused on the denaturation effect and cell viability after the HHP treatment for a fundamental study. To perform a basic validation for the proposed HHP-based decellularization method, cell suspensions and collagen gels were subjected to HHP with supercooling pretreatment. Furthermore, cell viability and preservation of the collagen gel structures were evaluated.

## 2. Materials and Methods

### 2.1. NB1RGB Cell Culture

In this study, the effect of HHP combined with supercooling pretreatment on normal human skin fibroblasts (NB1RGB, Riken BRC, Tsukuba, Japan) was evaluated to validate a decellularization method using low-level HHP with supercooling pretreatment. As reported in our previous study [32], NB1RGB cells were cultured in alpha-modified Eagle minimum essential medium (MEM α, Thermo Fisher Scientific Inc., Waltham, MA, USA), supplemented with 10% fetal bovine serum (FBS, Sigma-Aldrich, St. Louis, MO, USA) and 1% antibiotic–antimycotic (Nacalai Tesque, Kyoto, Japan) in a humidified CO_2_ incubator (5% CO_2_ at 37 °C). NB1RGB cells were suspended in MEM α culture medium without phenol red, supplemented with 10% FBS and 1% antibiotic–antimycotic to a concentration of 5.0 × 10^5^ cells/mL. The cell suspensions were subjected to supercooling pretreatment and HHP application. After treatment, the cell viability and proliferation were evaluated using a live/dead assay using calcein-AM and PI staining. Cell morphology was evaluated by scanning electron microscope (SEM).

### 2.2. Supercooling Device for Pretreatment of Cell Suspension

**Figure 1:**
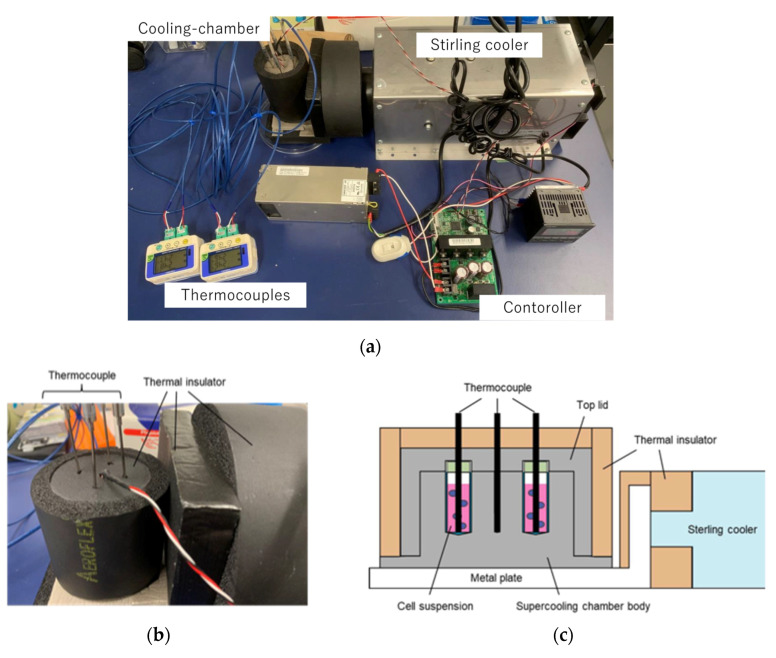
Schematic of supercooling device for cell suspension and collagen gel specimens. (**a**) The supercooling device consists of a supercooling chamber, thermocouples, a controller, and a Stirling cooler. (**b**) The supercooling chamber was made of aluminum and covered by thermal insulator materials. (**c**) Cryovials containing cell suspension were held in the chamber and the temperature of each cryovial was monitored by thermocouples.

In this study, supercooling pretreatment was performed on cell suspensions to denature cell membranes to improve the decellularization effect of HHP on normal human skin fibroblasts. A supercooling device for biological cells and tissues was developed to impose a cell suspension in a supercooling state. The supercooling device consists of a cooling chamber to hold and cool the cryovials and thermocouples to monitor the temperature inside the cryovials and cooling chamber, respectively, to control the temperature of the specimens (Figure 1a). The chamber was cooled using a Stirling cooler (SC UE15R, Twinbird Corp., Tsubame, Japan) with a temperature controller. The supercooling chamber was made of aluminum because of its excellent thermal conductivity. The supercooling chamber is divided into a main body and a top cover to hold four 1.2 mL cryovials (Figure 1b,c). A thermocouple was inserted into the center of the chamber for temperature control inside the cooling chamber. Other thermocouples were inserted through the top cover of the chamber to monitor the temperature inside the cryovials. The chamber was insulated from the surrounding environment to prevent the supercooling state from dissipating. The chamber was also connected to the heat-absorbing part of the Stirling cooler through an aluminum plate. The specimens were cooled under precise temperature control using our supercooling device.

The supercooling conditions of the specimens were set at −4 °C and −8 °C for 12 h, referring to our previous report on the effect of supercooling on cell viability and proliferation [32]. The maintenance of the supercooling condition was confirmed by the temperature history of the specimens, which showed that the temperature (i.e., −4 °C or −8 °C) never increased to 0 °C during the supercooling treatment (Figure S1).

### 2.3. High Hydrostatic Pressure Application with Supercooling Pretreatment to NB1RGB Cell Suspension

**Figure 2:**
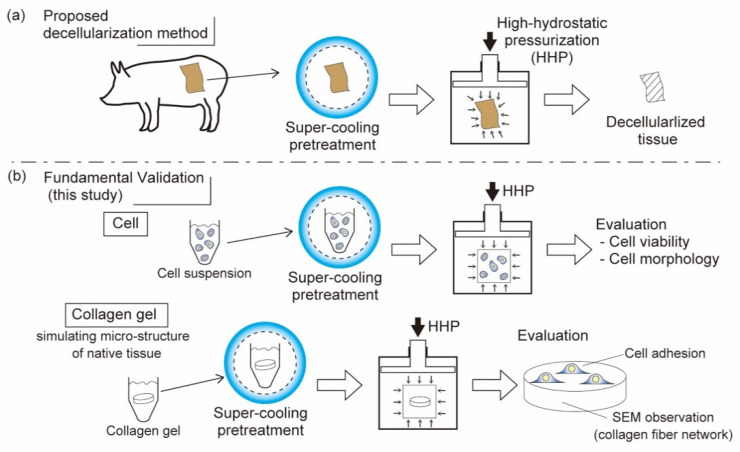
Proposed decellularization method using HHP with supercooling pretreatment. (**a**) Basic validation process of the decellularization method. (**b**) Effect of HHP with supercooling pretreatment on cell viability and morphology in cell suspension (upper part) were evaluated. Preservation of microstructure and cell adhesiveness of collagen gel simulating that of biological tissues (bottom part) were evaluated.

In this study, we established a pretreatment method of cells and biological tissues in a supercooled state to achieve physical decellularization by applying moderate and static HHP (Figure 2a). To validate our proposed method, we applied static and moderate HHP to cell suspensions after supercooling pretreatment (Figure 2b). The effects on cell viability and structure of the cell membrane were evaluated. The cell viability was evaluated using a live/dead assay using calcein-AM and PI staining. Structure of cell membrane was evaluated by scanning electron microscope (SEM).

**Figure 3:**
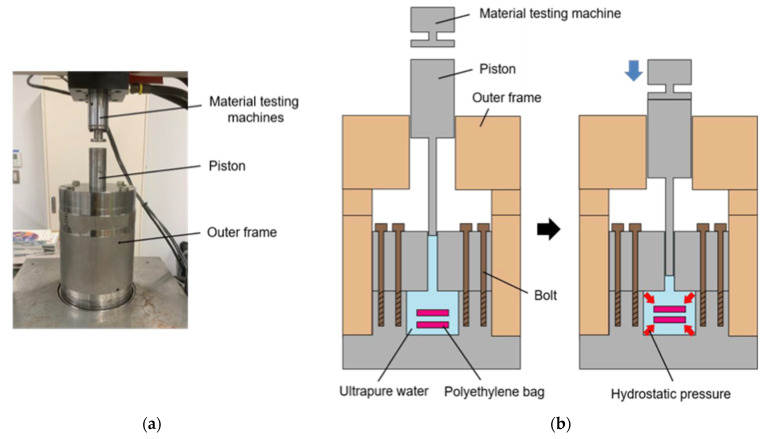
Schematic of device for high hydrostatic pressure application (HHP). (**a**) A piston direct pressurization vessel compressed by a material testing machine. (**b**) Polyethylene bags containing cell suspension or collagen gel were subjected to HHP by the piston pressurization system.

For the HHP application device, we used the device reported in our previous study [31]. The device consists of a piston direct pressurization vessel compressed by a material testing machine (MTS bionix858, MTS, Eden Prairie, MN, USA). HHP was applied to the cell suspension in the vessel by inserting a piston into the pressure vessel (Figure 3). The device could impose HHP on cell suspensions or biological tissues to reach a maximum HHP of 250 MPa and keep static HHP to control the compressive force monitored by the material testing machine.

For HHP application experiments combined with supercooling pretreatment, cell suspensions of human dermal fibroblasts (NB1RGB, Riken Cell Bank) were used as the specimen. NB1RGB cells were cultured in MEM + 10% FBS + 1% antibiotic–antimycotic. Cultured cells were detached and collected using 0.25% trypsin/EDTA solution to prepare the cell suspension in fresh medium without cryoprotectants to make a concentration of 1.0 × 10^6^ cells/mL. For the supercooling pretreatment, 1.2 mL of the cell suspension was injected into a cryovial and inserted into the precooled chamber at 20 °C. The cell suspension in the cryovial was then cooled to the supercooled state, which was maintained for 12 h. The supercooling temperatures were set at −4 °C and −8 °C, based on our previous study [32]. After this pretreatment, 600 μL of the cell suspension was dispensed from the cryovials into zippered plastic pouches for HHP decellularization treatment.

Following pretreatment by supercooling, HHP was applied to the cell suspensions. For static application of moderate HHP, the pressure was increased from 0 to 100, 150, or 200 MPa and held for 10 min. To suppress the damage of pressure vessel by HHP, the pressure was increased at a rate of 2.68 MPa/s from 0 to 150 MPa and 1.68 MPa/s from 150 to 200 MPa during pressurization. The number of specimens was *n* = 4, consisting of four conditions: no hydrostatic pressure, 100, 150, and 200 MPa for 10 min. Three pretreatment conditions, untreated, refrigerated, and supercooled, were used to investigate the effect of the supercooling pretreatment. The experimental conditions are presented in Table 1.

Cell viability was evaluated by fluorescence microscopy with calcein-AM and PI staining. The cytoplasm of living cells could be stained by calcein-AM, the cell nuclei of dead cells could be stained by PI. For calcein-AM/PI staining, NB1RGB cells were subjected to washing with phosphate-buffered saline (PBS), following with incubation with 0.1 mg/mL calcein-AM and PI in PBS for 10 min. To characterize the live/dead cell ratio, cell numbers of cells stained with calcein-AM and PI were counted. Cell morphology and structure of cell membrane were evaluated by environmental scanning electron microscopy (E-SEM). The cells subjected to HHP with supercooling pretreatment were fixed in a 4% paraformaldehyde-phosphate buffer solution (Nacalai Tesque, Kyoto, Japan) for 30 min. The fixed cells were treated with 25, 50, 75, 90 and 99.5% ethanol dehydration. After dehydration, the cells were observed using an E-SEM with an accelerating voltage of 5.0 kV (Inspect S50, FEI, Tokyo, Japan).

### 2.4. High Hydrostatic Pressure Application with Supercooling Pretreatment to Collagen Gel Scaffold

In the decellularization of biological tissues, it is necessary to remove only the cells and maintain the microstructure of ECM. In this study, collagen gels were subjected to HHP with supercooling pretreatment to examine the effect of our decellularization method on the microstructure and function of ECMs (Figure 2b).

Collagen gels were prepared from a type I collagen neutral solution. The collagen neutral solution was prepared at a final concentration of 2.4 mg/mL from acid-soluble collagen (I-AC30, KOKEN, Tokyo, Japan). The collagen solution (0.3 mL) was poured into 24-well plates and incubated in a CO_2_ incubator for 30 min. The gels were subjected to decellularization treatment under the same conditions as mentioned in Section 2.4. The treated collagen gels were placed in 48-well plates to cover the whole surface of each well and to culture 1.0 × 10^4^ cells/well of NB1RGB cells for 12 h to evaluate the effect of HHP with supercooling pretreatment on cell adhesiveness of collagen gel. After culturing, the cells were stained with calcein-AM to observe the morphology of the adhered cells. The cell staining and microscopic observation methods were the same as those described in Section 2.4. To evaluate cell number attached to the collagen gel, the total DNA content of cells adhered on collagen gel was evaluated using the fluorometric DNA assay [33,34,35]. The cells attached to the collagen gel surface were solubilized with 125 μg/mL papain (Sigma-Aldrich, St. Louis. MO, USA) solution at 60 °C for 6 h, and the total DNA content of cells in one well was quantified using a fluorescence spectrophotometer (Qubit 2.0 Fluorometer, Life Technologies, Carlsbad, CA, USA). The number of cells was proportional to the total DNA content of cells [33], and the number of adhered cells on the collagen gels could be evaluated.

### 2.5. Statistical Analysis

Most of the experimental data are representative of three individual experiments with similar results. For each group, four samples (*n* = 4) were evaluated, and each data point indicated the mean and standard deviation. The statistical significance of the data was evaluated using the Tukey–Kramer method. Statistical significance was set at *p* < 0.05.

## 3. Results

### 3.1. Effect of Single HHP Exposure with Supercooling Pretreatment on Cell Suspension

**Figure 4:**
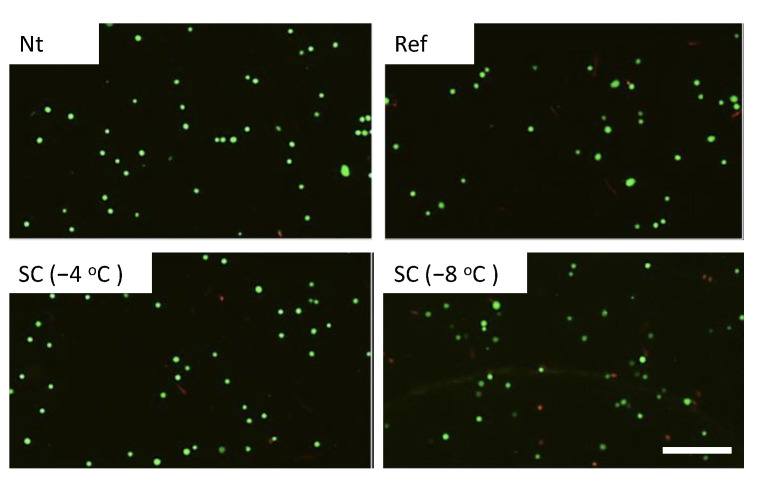
Fluorescent images of calcein-AM (green) and PI (red) for cell suspension without HHP application. Living cells were stained by calcein-AM and dead cells were stained by PI. The scale bar indicates 200 μm.

**Figure 5:**
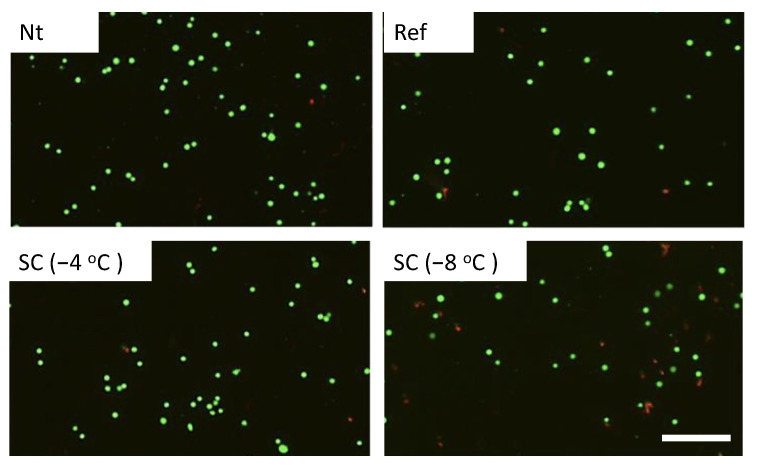
Fluorescent images of calcein-AM (green) and PI (red) for cell suspension after HHP application of 100 MPa with different pretreatments. The scale bar indicates 200 μm.

**Figure 6:**
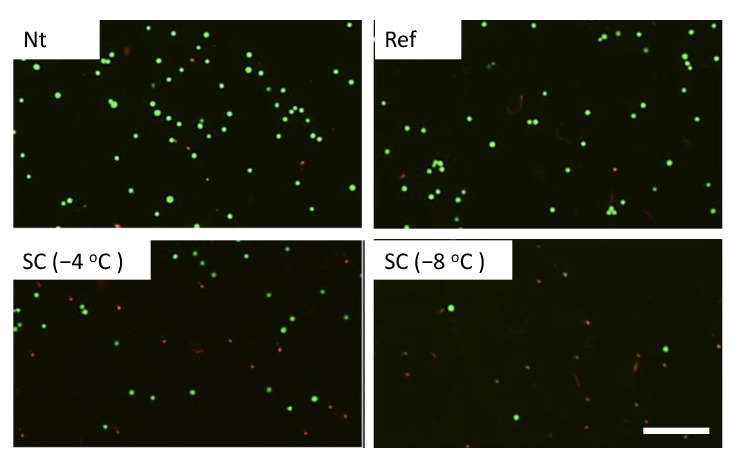
Fluorescent images of calcein-AM (green) and PI (red) for cell suspension after HHP application of 150 MPa with different pretreatments. The scale bar indicates 200 μm.

**Figure 7:**
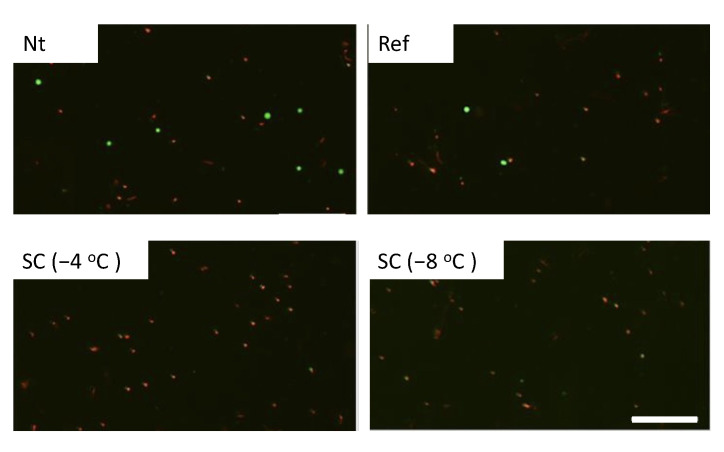
Fluorescent images of calcein-AM (green) and PI (red) for cell suspension after HHP application of 200 MPa with different pretreatments. The scale bar indicates 200 μm.

Calcein-AM and PI staining revealed that cell viabilities were about 90% in the experimental groups without pretreatment, under pretreatment by refrigeration at 4 °C, and supercooling at −4 °C in the case of no HHP treatment (Figure 4). In the case of HHP treatment at 100 MPa, cell viabilities were also about 90% in the experimental groups except for the group under pretreatment by supercooling at −8 °C (Figure 5). In contrast, the number of viable cells significantly decreased in the experimental group that was subjected to supercooling pretreatment at −8 °C. In the case of the HHP treatment at 150 MPa, cell viabilities were about 90% in the experimental groups without pretreatment and under pretreatment by refrigeration at 4 °C (Figure 6). In contrast, the number of viable cells significantly decreased in the experimental group that was subjected to HHP at 150 MPa under supercooling pretreatment. In the case of the 200 MPa HHP treatment, the cell number of all experimental groups decreased after HHP application (Figure 7).

**Figure 8:**
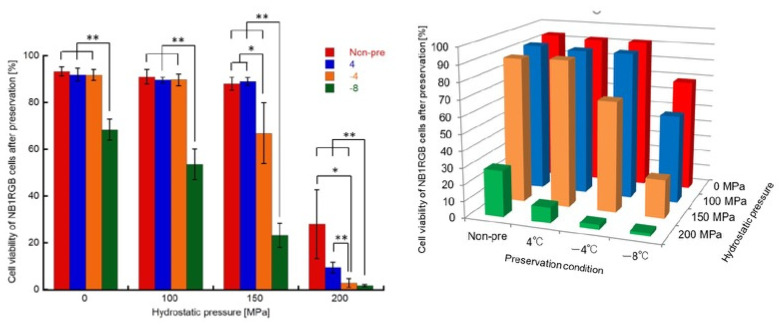
Cell viability after the HHP application with different pretreatment. In the case of HHP treatment at 200 MPa, the percentage of viable cells with the supercooling treatment was reduced to 2–3% of non-treated cells. Data are presented as mean ± S.D, *n* = 4 * and ** indicate significant differences as compared to the value of Nt group (*: *p* < 0.05, **: *p* < 0.01).

The percentages of viable cells in the experimental groups treated with HHP at 200 MPa under all pretreatment conditions were lower than those with HHP at 150 MPa (Figure 8). In particular, the percentage of viable cells with the supercooling treatment was reduced to 2–3% of non-treated cells by 200 MPa of HHP treatment.

**Figure 9:**
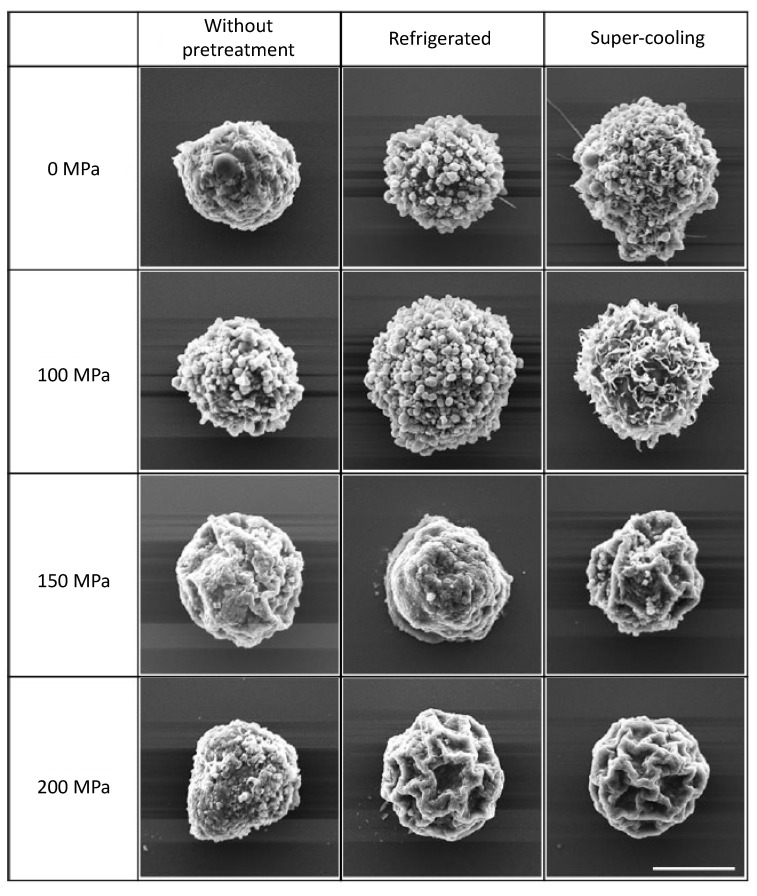
SEM images of NB1RGB cells after HHP application after various pretreatment. The application of HHP at with pretreatment by refrigeration and supercooling resulted in folding of cell membranes and cell shrinkage, similar to the result under the HHP application at 150 MPa with supercooling pretreatment. Scale bar: 5 μm.

SEM observations were performed to evaluate the cell microstructure subjected to HHP with supercooling pretreatment (Figure 9). In the experimental groups without any pretreatment, the microstructure of the cell membrane was maintained under all HHP application conditions, and no significant degeneration of the cell membrane was observed. No change in cell membranes was observed in the experimental groups pretreated with refrigeration and supercooling without HHP application. No degeneration of the cell membrane was also observed under HHP application at 100 MPa with any pretreatment condition. No significant degeneration was observed in the membrane of cells subjected to HHP at 150 MPa without pretreatment and with refrigeration pretreatment, whereas the membrane of cells subjected to HHP at 150 MPa with supercooling pretreatment was folded and contracted. In the HHP at 200 MPa treatment group, the application of HHP with pretreatment by refrigeration and supercooling resulted in the folding of cell membranes and cell shrinkage, similar to the result under the HHP application at 150 MPa with supercooling pretreatment.

### 3.2. Effect of Static HHP Exposure with Supercooling Pretreatment on Micro Structure of Collagen Gel

In the decellularization process, only the cells should be removed from the living tissue, while the microstructure of the ECM and the structure of tissues and organs that maintain the function of the living body must be preserved. In this study, the effect of HHP with supercooling pretreatment on the microstructure and function of collagen gel material was evaluated.

**Figure 10:**
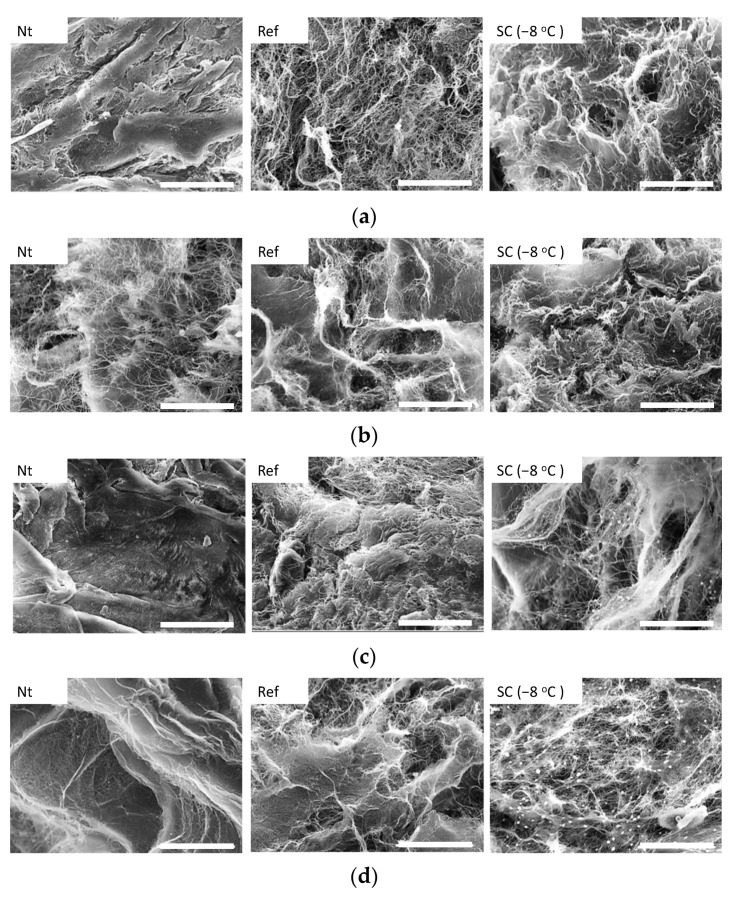
SEM images of collagen gel (**a**) without HHP application, and with HHP application of (**b**) 100 MPa, (**c**) 150 MPa, and (**d**) 200 MPa. In all experimental groups, there was no breakage or aggregation of collagen fibers. The scale bar indicates 20 μm.

**Figure 11:**
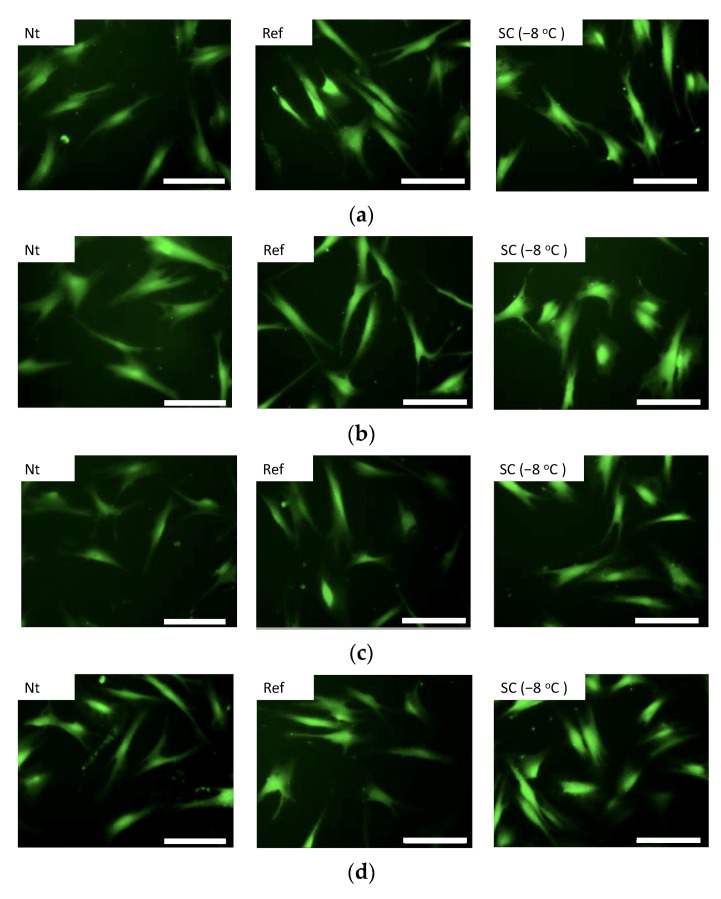
Fluorescence images of calcein-AM staining of NB1RGB cells. The cells adhered and extended their pseudopodia on collagen gels in all experimental groups. The gels were treated (**a**) without HHP and with HHP application of (**b**) 100 MPa, (**c**) 150 MPa, and (**d**) 200 MPa. The scale bar indicates 100 μm.

**Figure 12:**
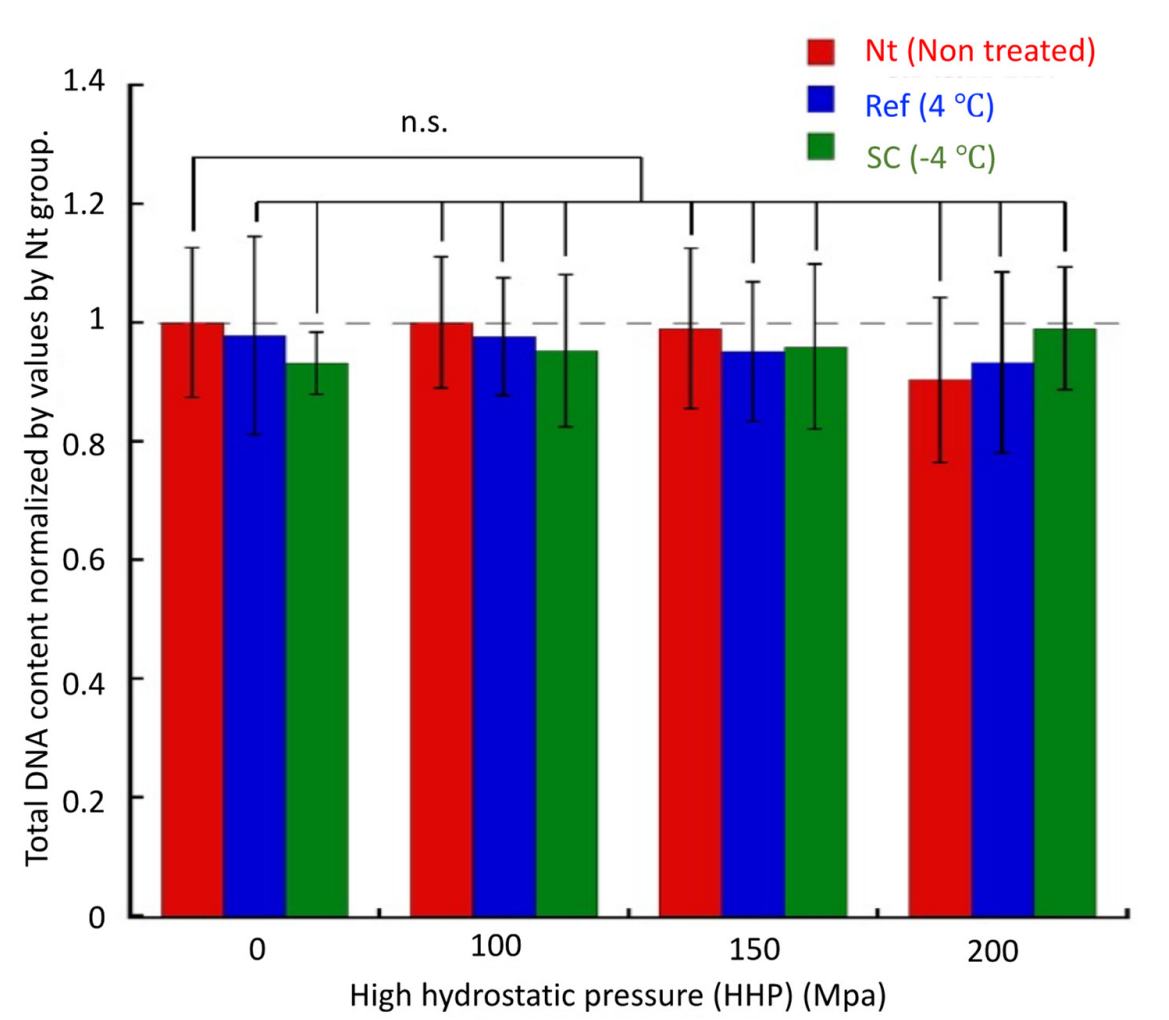
Total DNA content of NB1RGB cells adhered on collagen gels subjected to HHP with different pretreatments. There was no significant difference in the total DNA content among all experimental groups. Mean ± S.D., *n* = 4.

SEM observations were performed to evaluate the microstructure of the collagen gel after HHP exposure with supercooling pretreatment (Figure 10). In all experimental groups, there was no breakage or aggregation of collagen fibers, and there was no denaturation of collagen by application of HHP application with pretreatment by supercooling. The NB1RGB cells seeded on the surface of collagen gels grew and extended their pseudopodia on the gels in all experimental groups (Figure 11). The total DNA content, that is, the number of cells in all specimens after 12 h of culture, showed similar values. There was no significant difference in the total DNA content (i.e., the cell number) among all the specimens after 12 h of culture (Figure 12).

## 4. Discussion

In this study, the effect of supercooling pretreatment followed by HHP application on a NB1RGB cell suspension and collagen gel scaffold material was examined to assess the hypothesis that the supercooling treatment would denature and weaken the cell membrane in order to improve the efficacy of HHP-based decellularization, without destruction or degeneration of ECMs. After a single HHP application with supercooling pretreatment, the viability of cells subjected to over 150 MPa of HHP decreased as compared to the single HHP application.

Frey et al. reported that the viability of eukaryotic cells subjected to HHP was not significantly reduced below 150 MPa [36]. Diehl et al. investigated the survival rate of human fibroblasts subjected to high hydrostatic pressure and reported that 50% of cells were killed when 130–145 MPa of HHP was applied [37]. These previous reports indicated that cell viability could be maintained at an HHP of approximately 150 MPa. In our previous study, we found that cell viability was not reduced by static HHP application at 150 MPa [31]. In contrast, the combination of the supercooling pretreatment proposed in this study could reduce the cell viability to approximately 20%, even with HHP treatment at 150 MPa. It was considered that the supercooling pretreatment made the cell membranes fragile and improved the efficiency of cell destruction by HHP application. Moreover, static and moderate HHP application at 200 MPa successfully reduced the cell viability to 2–3% in both −4 °C and −8 °C supercooling pretreatments. In our previous study, we reported that cyclic HHP application at 250 MPa reduced cell viability to approximately 20% and caused denaturation of cell membrane [31]. The efficiency of our proposed method was superior to the reduction ratio of cell viability in our previous study and also superior to that in other physical decellularization methods using static and moderate HHP [36,37].

In our previous study, we reported that the supercooling of cell suspension without cryoprotectants reduced cell viability and the adhesive and proliferative abilities of surviving cells after supercooling treatment [32]. In the present study, the SEM images showed that the cell membranes were folded and cells were contracted in the experimental groups subjected to HHP with supercooling treatment. This phenomenon was caused by the weakening and flexibility of the cell membrane due to the supercooling pretreatment, which led to the destruction of the cell membrane and cell death.

In the decellularization process of biological tissues, it is important to preserve the microstructures of tissues and improve the removal efficiency of cells from tissues. To validate that the proposed decellularization method preserves the microstructure of tissues, we applied HHP to collagen gels simulating biological tissues with supercooling pretreatment. The NB1RGB cells were cultured on the collagen gel treated by HHP with supercooling pretreatment, and the cells grew and extended their pseudopodia under all experimental conditions. In addition, the SEM images showed that the collagen fiber structure was not damaged by any treatment. Nan et al. reported that an ultrahigh hydrostatic pressure of 500 MPa has a risk of denaturing collagen network structures [38]. Our proposed HHP treatment with supercooling pretreatment is expected to be an efficient method for physical decellularization to preserve the microstructures of biological tissues.

In this study, we demonstrated that decellularization could be achieved with preserving the microstructure of tissues by applying HHP with supercooling pretreatment. To apply our decellularization method in clinical settings, validation studies using native organs or tissues are required. We expect that, in future studies, our proposed method will produce decellularized tissues that preserve the microstructure of biological tissues through verification using native tissues and organs.

## 5. Conclusions

In this study, the effects of supercooling pretreatment followed by moderate HHP application on the microstructure of living cells and collagen gel materials were evaluated. Our experimental results suggested that supercooling pretreatment promoted the denaturation and weakening of the cell membrane to improve the efficacy of decellularization using static application of moderate HHP. Furthermore, it was demonstrated that the HHP with supercooling pretreatment did not degenerate and damage the microstructure of the collagen gel. It is expected that our novel decellularization method using moderate HHP application with supercooling pretreatment can be employed to manufacture decellularized tissue more effectively without degeneration of the ECM in living tissues.

## Figures and Tables

**Table 1:** Experimental conditions for HHP application with supercooling pretreatment.

Experimental Groups	Pretreatment	HHP
Nt	Non-treated	0, 100, 150, 200 MPa for 10 min
Ref	Refrigerated at 4 °C	
SC (−4 °C)	Supercooled at −4 °C	
SC (−8 °C)	Supercooled at −8 °C	

## Supplementary Materials

The following are available online at https://www.mdpi.com/article/10.3390/mi12121486/s1, Figure S1: Time course of temperature during supercooling treatments at (a) −4 °C and (b) −8 °C. The temperature never increased to 0 °C during the supercooling treatment.
