# Supplementary material for: Efficient Decellularization by Application of Moderate High Hydrostatic Pressure with Supercooling Pretreatment
Source: Micromachines (Basel). 2021 Nov 30;12(12):1486. doi: 10.3390/mi12121486 (PMC8708072; doi:10.3390/mi12121486)
Supplement: Supplementary file 1 [file micromachines-12-01486-s001.zip › micromachines-1453070-supplementary.pdf]

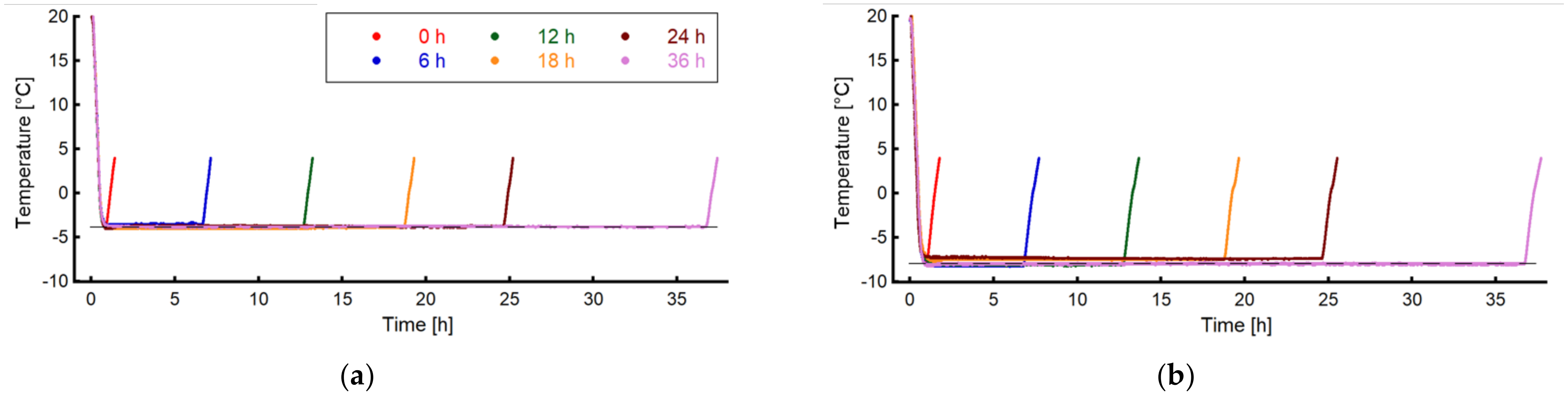

Figure S1. Time course of temperature during supercooling treatments at (**a**) -4 °C and (**b**) -8 °C . The temperature never increased to 0 °C during the supercooling treatment
